# A QM/MM Derived Polarizable Water Model for Molecular Simulation

**DOI:** 10.3390/molecules23123131

**Published:** 2018-11-29

**Authors:** Koen M. Visscher, William C. Swope, Daan P. Geerke

**Affiliations:** 1 AIMMS Division of Molecular Toxicology, Department of Chemistry and Pharmaceutical Sciences, Faculty of Science, De Boelelaan 1108, 1081 HV Amsterdam, The Netherlands; k.m.visscher@vu.nl; 2 IBM Almaden Research Center, 650 Harry Road, San Jose, CA 95120, USA; swope@us.ibm.com

**Keywords:** molecular dynamics simulations, polarizable force field, QM/MM calculations, higher-order dispersion, charge-on-spring model, water

## Abstract

In this work, we propose an improved QM/MM-based strategy to determine condensed-phase polarizabilities and we use this approach to optimize a new and simple polarizable four-site water model for classical molecular simulation. For the determination of the model value for the polarizability from QM/MM, we show that our proposed consensus-fitting strategy significantly reduces the uncertainty in calculated polarizabilities in cases where the size of the local external electric field is small. By fitting electrostatic, polarization and dispersion properties of our water model based on quantum and/or combined QM/MM calculations, only a single model parameter (describing exchange repulsion) is left for empirical calibration. The resulting model performs well in describing relevant pure-liquid thermodynamic and transport properties, which illustrates the merit of our approach to minimize the number of free variables in our model.

## 1. Introduction

The presence of water is vital for biomolecular action and cellular function [1,2]. It is therefore necessary to simulate systems in a water model that can accurately mimic solvent environmental effects when studying e.g., protein dynamics and ligand binding [3,4,5]. Due to its diverse properties and counter-intuitive behavior including a density maximum at 4 °C, calibrating simple but well-performing atomistic models for water is far from trivial [6,7,8]. First-generation water models commonly used in molecular simulation have three interaction sites, with static partial charges located on the oxygen and both hydrogens [9,10] and with dispersion and exchange interactions handled by a single van der Waals site per water molecule (at the oxygen). A limitation of models with three interaction sites is that they poorly describe higher-order multipole moments of the water molecules. The molecular dipole and quadrupole moment are partially due to a shift of electron density from the hydrogens to the more electronegative oxygen atom. Simple atomistic models with a fourth off-atom interaction site were introduced in order to more accurately describe electrostatic molecular properties, which simultaneously improved the description of energetic and structural pure-liquid properties [9,11,12,13]. In this way, models became available that correctly describe the melting temperature of water, whereas previous generations of water models failed to describe this phenomenon [14,15]. While four-site static-charge models can describe structural and energetic properties well, they typically underestimate electrostatic screening expressed through the static dielectric permittivity [14,15].

The difficulty in describing dielectric screening in simulation can be addressed by calibration of explicitly polarizable models, in which the response of induced dipole moments to external electric fields is modeled e.g., by movable massless charges attached via a spring to the polarizable nucleii [7,8,12,13,16]. Polarizable models can accurately describe molecular properties at ambient temperatures, but many of these models still have difficulties in reproducing experimental values for the melting temperature and show a constant increase in density with decreasing temperature [7,16,17]. In contrast, the AMOEBA model which includes a next level of detail into its polarizable force field parameter sets (i.e., static atomic dipole and quadrupole moments) has been successful in describing water properties over a range of state points [18,19]. In the current work, we reevaluate the use of a simple four-site polarizable model to describe water properties in classical simulation. As a starting point, we reconsider the parameterization of van der Waals dispersion interactions in the model. This is motivated by the recent work of Mohebifar et al., who showed that molecular values for the $C_{6}$ dispersion parameter are typically significantly too high in molecular mechanics (MM) models when compared to reference quantum estimates [20], whereas Shaw and co-workers found evidence that the reference value for water would in turn be too low to describe solvent dispersion in simulations of disordered proteins [21,22]. Recently, we showed that, by introducing a higher-order dispersion term ($C_{8}$, which can contribute up to one third of molecular dispersion interactions) and by using atoms-in-molecules (AIM) quantum calculations of Exchange-Hole-Dipole moments (XDM) to determine van der Waals parameters [23], we obtained an alkane model that reproduces pure-liquid thermodynamic properties within a few percent without further parameter calibration [24]. In the current work, we investigate the performance of a simple (and rigid) four-site polarizable water model with a single van der Waals and polarizable center, in which we introduce higher-order dispersion via a $C_{8}$ term as well. (Molecular) $C_{6}$ and $C_{8}$ values will be assigned from XDM calculations [20,23], and static partial charges of the off-atom site and hydrogen atoms as well as bond lengths are also directly determined from quantum calculation.

To compute a model value for the polarizability and as major part of our current efforts, we design and use here an adapted version of our recently proposed approach to determine condensed-phase polarizabilities from combined QM/MM calculations [25,26]. Previously, we were successful in fitting atomic polarizabilities for small alcohols from distributions of QM/MM-derived values, as obtained for snapshots of solvent configurations around a given solute [25]. However, relatively wide distributions were obtained which led to uncertainties in the fitted polarizabilities [25,26]. As a remedy, we propose here a constrained-fitting strategy and demonstrate that our redesigned approach allows for determining a consensus value for the polarizability based on our QM/MM calculations. Together with the other parameters derived from quantum (and XDM) calculations, this leaves us with a single parameter (i.e., the repulsive van der Waals constant) to be empirically calibrated in order to obtain our final water model. We calibrate this parameter based on pure-liquid thermodynamic properties of water at ambient conditions, and we find that our final model has a static dielectric permittivity at room temperature and heat of vaporization at a wide range of temperatures (250–370 K) that are close to experimental estimates, while it also shows a maximum in water density in the expected region.

## 2. Methods

### 2.1. Computational Details for QM/MM Based Polarizability Fitting

**Figure 1:**
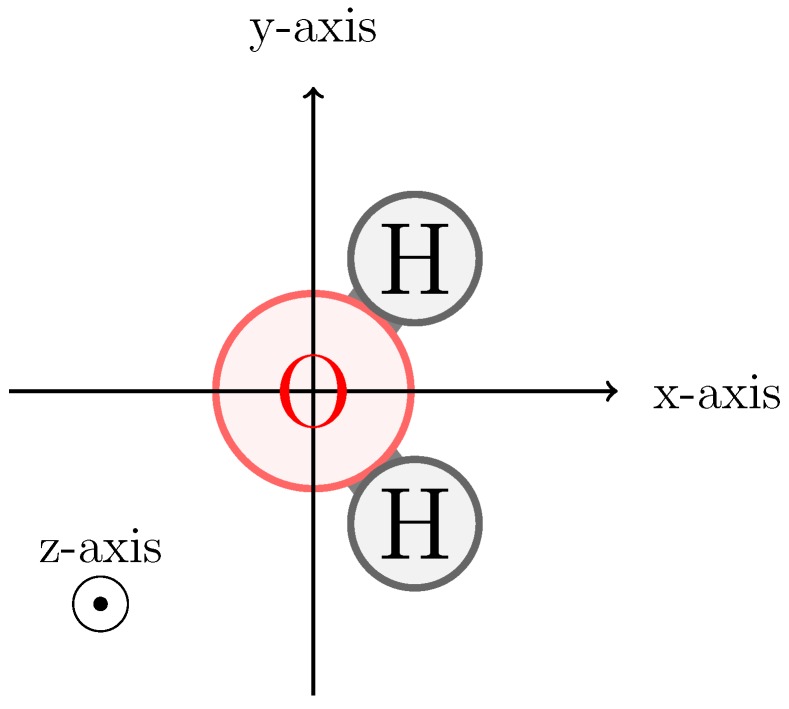
Axis alignment of the QM water solute as applied during polarizability and charge fitting. The oxygen is placed at the origin of the axis system and the hydrogens are placed in the positive *x*-axis regime. Only the sign of the *y*-axis value differentiates between the first and second hydrogen. The molecule is placed in the *x*–*y* plane and the *z*-axis is defined as *x* × *y*.

To fit effective atomic polarizabilities, we use an extension of our previously published QM/MM protocol [25]. In this protocol, a query (solute) compound is first geometry optimized in vacuo at the B3LYP/QZ4P level of theory using the Amsterdam Density Functional (ADF) package and subsequently aligned along the *x*-axis (Figure 1) [27,28,29].

The resulting geometry is solvated in a cubic box of 28.4 nm^3^ filled with pre-equilibrated SPC water (1000 SPC molecules in this work) [10]. Note that use of different solvent models was previously shown to lead to similar QM/MM fitted values for the polarizability [25,26]. In the subsequent energy minimization and molecular dynamics (MD) simulations, the solute is positionally constrained to the QM-optimized geometry in vacuum (aligned according to Figure 1). The leap-frog algorithm is used during MD to integrate Newton’s equations of motions using a time step of 2 fs. Following a steepest-descent energy minimization, the simulation system is equilibrated during 100 ps under *NVT* conditions. Production simulations lasted for 2 ns under *NVT* conditions, where coordinates are written out every 4 ps (500 frames). The temperature was weakly coupled to an external bath using a Berendsen thermostat with a coupling constant of 0.1 ps and a target temperature of 298.15 K [30]. Interactions were handled using a twin-range pairlist with a short-range cutoff of 0.8 nm updated every time step and a long-range cutoff up to 1.4 nm, where interactions are updated every fifth time step. A reaction field long range correction was added [31,32], with a cutoff distance of 1.4 nm equal to the long range cutoff ($r_{c,rf}=r_{c,lr}$). The homogeneous medium outside of the cutoff was assigned a dielectric constant of 78.4, equal to the experimental value for water [33]. The SPC molecules in the simulations were kept rigid and were constrained using SHAKE with a relative tolerance of 0.0001 [34]. From each of the 500 frames, a unique set of solvent coordinates is extracted and gathered around the solute molecule. Only water molecules of which the oxygen is within an interaction distance (1.4 nm) of any of the solute atoms are considered for the combined QM/MM calculations. The point charges from the considered water molecules are introduced as Bq (i.e., MM partial) charges in a quantum calculation at the B3LYP/QZ4P level of theory. These QM/MM calculations are used to evaluate effective electrostatic potentials (ϕ) at grid points around the solute molecule. A suitable Connolly grid [35,36,37] for this analysis is generated using GAMESS-US 2014 [38,39], using four incremental layers with a point density of five points per bohr^2^ as described in detail in Ref. [25].

### 2.2. Electrostatic Model

Static partial charges were fitted to reproduce the QM-determined vacuum electrostatic potential of the water solute molecule, used as reference during the polarizability calculations. A static charge was placed on both hydrogens and on an off-atom M-site placed at 0.0225 nm distance from the oxygen atom on the *x*-axis in the direction of the hydrogen atoms (cf. Figure 1). The magnitudes of the static charges were determined in a least-squares fitting protocol with an additional constraint for the total molecular charge to be zero [40].

Explicit polarization was treated using the Charge-On-Spring method (COS) [13,17,41,42], where a weightless movable charge is attached to polarizable sites. In the case of our simple four-site water model, this movable charge is attached to the off-site (M) with a polarizability as determined using our QM/MM protocol. The COS particle was assigned a charge of −8.0 e, consistent with previous work by us and others, as this value allows for the determination of electric fields on the attached site and not the COS itself (which is more expensive) [8,13,17,43]. The positions of these COS particles are updated in a self-consistent field optimization (SCF) between steps in the simulation, consistent with a Born–Oppenheimer treatment of the system [44].

### 2.3. Dispersion Calculations

To compute molecular dispersion constants for water, Quantum Mechanical (QM) calculations of a single molecule were performed on the B3LYP level of density-functional theory using an augmented spherical basis set (aug-cc-pVTZ) [45,46]. Dispersion parameters were calculated using Exchange-Hole-Dipole moment calculations (XDM) as described in our previous work on dispersion calculations [24]. Molecular electron densities resulting from these QM optimizations are partitioned into atomic contributions using an iterative Hirshfeld scheme (Hirschfeld-I) [47] with pro-atom densities that were computed at the same B3LYP/aug-cc-pVTZ level of theory. Note that the sum of these atomic densities reproduces exactly the molecular electron density [48]. The atomic partitioning was performed in Horton version 2.1.0 [49], and both the integration grid and global Hirshfeld weights for each atomic center were written out to file. A modified version of the postg program was used to compute the exchange-hole-dipole moments and to translate these into atomic $C_{6}$ and $C_{8}$ dispersion coefficients [50,51,52,53]. At this time higher order ($C_{10}$ and up) dispersion coefficients are not used. In our previous alkane work in which we introduced higher order dispersion parameters into molecular simulation, $C_{11}$ was found to be the most optimal repulsive shape to include Pauli exclusion effects [24]. The resulting van der Waals interaction is a $C_{6}$-$C_{8}$-$C_{11}$ potential where the $C_{6}$ and $C_{8}$ are attractive terms and the $C_{11}$ is a repulsive term (Equation (1)). Note that while the choice of $C_{11}$ was empirical and based on tests on pure-liquid simulations, a $C_{11}$ repulsive term is a natural choice considering that, physically, the odd dispersion terms are repulsive in nature [54]:

(1) $$V_{vdw}=-\frac{C_{6}}{r^{6}}-\frac{C_{8}}{r^{8}}+\frac{C_{11}}{r^{11}}.$$

### 2.4. Pure-Liquid MD Simulations

Pure-liquid simulations were performed for all water models with GROMOS11 md++ version 1.4.0 [55,56], with a modified van der Waals potential function to accommodate explicit $C_{8}$ attractive and $C_{11}$ repulsive terms. For each simulation, 1024 water molecules were placed in a rectangular box using the GROMOS++ tool ran_box under cubic periodic boundary conditions [57]. Pure-liquid properties were inferred from *NpT* simulations in which the reference temperature was coupled weakly to an external bath using the Berendsen thermostat with a coupling time of 0.1 ps [30]. To discard possible artifacts [58] due to use of this thermostat, we compared our computed values for the density and heat of vaporization with those when using a Nosé–Hoover thermostat, which differed within 0.01% only. Pressure was maintained at 1 atm using a Berensden barostat with a coupling time of 0.5 ps [30]. Equations of motion were integrated with a time step of 2 fs and using the leap-frog algorithm. Van der Waals benchmark runs consist of a 100 ps thermalization process in five steps, followed by an equilibration procedure under production conditions for 500 ps and production runs were 250 ps in total. The final model was simulated for a total of 12 ns using a 2 ns pre-equilibration period. Coordinates were written out every 1 ps for subsequent analysis, while instantaneous energy components were written out at 0.2 ps resolution. The water model was geometrically constrained using three bond vectors and the SHAKE algorithm with a relative geometric tolerance of 0.0001 [34].

### 2.5. Pure-Liquid Property Analysis

For the determination of pure-liquid properties the GROMOS++ run analysis toolkit was used [57]. Pure-liquid properties were determined from the instantaneous energy components as written out by md++. Average densities were obtained through the calculation of the ratio between the total system mass (number of water molecules $N_{water}$ multiplied by the molecular mass $M_{water}$) and the average box volume *V* as depicted in Equation (2):

(2) $$\langle \rho \rangle =\frac{N_{water}\cdot M_{water}}{\langle V\rangle }.$$

The heat of vaporization ($\Delta H_{vap}\left(T\right)$) was calculated from the difference of the averaged molecular potential energy in the gas phase $\langle E_{gas}\rangle$ and the condensed phase $\langle E_{liq}\rangle$ with the addition of the ambient pressure work (Equation (3)). Two correction terms were added ($C_{vib}$ and $C_{ni}$) that account for the change in vibration modes and non-ideal gas behavior of water, respectively. Values for both correction terms at temperature intervals relevant to this work are tabulated by Horn et al. [14]:

(3) $$\Delta H_{vap}\left(T\right)=\langle E_{gas}\rangle -\langle E_{liq}\rangle +nRT+C_{vib}+C_{ni}.$$

To calculate the dielectric permittivity of the liquid, the box dipole response to a homogeneous external electric field is used (Equation (4)). The ratio of the response dipole in the z-dimension $\langle M_{z}\rangle$ for a box with volume *V*, with the external (z-based) electric field $E_{z}^{ext}$ determines the static dielectric permittivity ϵ(0) [14,59,60]:

(4) $$\epsilon \left(0\right)=1+4\pi \frac{\langle M_{z}\rangle }{VE_{z}^{ext}}.$$

Diffusion coefficients were calculated using the Einstein relation, where the self-diffusion coefficient is determined in the limit of the mean-square displacement (Equation (5)), where $\vec{r}\left(t\right)$ denotes the oxygen position at time *t*:

(5) $$D_{pbc}=\lim_{t\to \infty }\frac{\langle {\left(\vec{r}\left(\tau +t\right)-\vec{r}\left(\tau \right)\right)}^{2}\rangle }{6t}.$$

Diffusion coefficients were corrected for simulation box sizes using the method developed by Yeh and Hummer [61], where a diffusion correction is computed using the shear viscosity (η) of the system (Equation (6)):

(6) $$D=D_{pbc}+\frac{2.837297\;k_{B}T}{6\pi \eta L}.$$

The secondary properties isobaric heat capacity ($C_{p}$), thermal expansion coefficient ($\alpha_{p}$), shear viscosity (η) and isothermal compressibility ($\kappa_{T}$) were calculated exactly as described in Refs. [8] and/or [62].

## 3. Results and Discussion

To determine the value of the polarizability to be used in our water model, we started using our previously published protocol based on condensed-phase QM/MM calculations of a QM water solute surrounded by water solvent molecules described at the MM level [25,26]. For that purpose, we extracted 500 MD-generated configurations of water shells around the solute. These solvent shells were used to evaluate the solute’s molecular electrostatic potentials (MEPs) in a condensed-phase environment, evaluated on a Connolly grid surrounding the water solute [35,36,37]. We expect a difference between the solute’s MEP in the gas and condensed phase due to polarization of the solute. In our QM/MM calculations, the solute is polarized by introduction of the Bq (MM partial) charges of the surrounding solvent waters in the quantum calculation. The difference between the MEP (ϕ) on a grid point in a condensed-phase (solv) and gas-phase environment (vac) is given in Equation (7):

(7) $$\phi_{induced}=\phi_{solv}-\phi_{vac}.$$

The induced potential $\phi_{induced}$ can be represented by fitting induced atomic dipole moments at the polarizable centers of interest, Equation (8). Here, $\vec{\mu_{i}}$ denotes the induced dipole at polarizable center *i* that generates a potential toward a grid point *n* with connecting vector $\vec{r_{in}}$ (with norm $r_{in}$):

(8) $$\phi_{\mu ,n}=\sum_{i}\frac{1}{4\pi \epsilon_{0}}\frac{\vec{\mu_{i}}\cdot \vec{r_{in}}}{r_{in}^{3}}.$$

The model assumes that the $\vec{\mu_{i}}$’s are due to an external electric field $E_{i}$ at each polarizable center *i*. These external electric fields can be computed using a simple Coulomb model (Equation (9)) that sums over the influence of all external (MM) point charges $q_{j}$:

(9) $$E_{i}=\sum_{j}\frac{1}{4\pi \epsilon_{0}}\frac{q_{j}\;\vec{r_{ij}}}{r_{ij}^{3}}.$$

**Figure 2:**
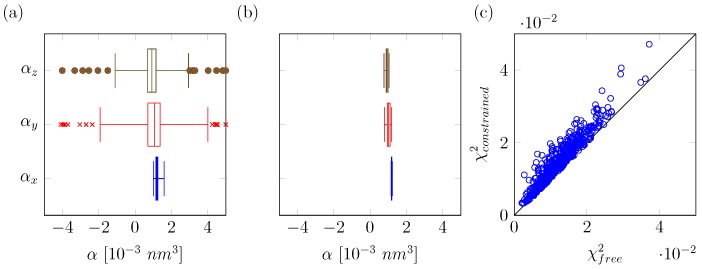
(**a**) box plots of the calculated polarizabilities in either *x*-, *y*- or *z*-dimension using the original fitting method per solvent configuration; (**b**) box plots of the calculated polarizabilities in either *x*-, *y*- or *z*-dimension using the new consensus fitting approach for polarizabilities. In panels (**a**,**b**), data are partitioned after sorting into four quartiles, and boxes depict the inter-quartile-range (irq) with a middle line that denotes the median. The whiskers are placed at the minimum and maximum values considered, with a maximum deviation of 4.0 times the irq. Outliers are denoted by circles (for $\alpha_{z}$) and crosses (for $\alpha_{y}$); (**c**) error in fit ($\chi^{2}$) as obtained from the free unconstrained original fit (free) and compared to the constrained consensus-fit error in fit.

To determine polarizabilities at center *i*, our original approach computes the ratio between the fitted induced dipoles and known electric fields for each of the diagonal components of the polarizability tensor ($\alpha_{i,xx}$, $\alpha_{i,yy}$ and $\alpha_{i,zz}$). In our previous work, we were successful in deriving effective polarizabilities for molecular simulation by averaging over the medians of the distributions of $\alpha_{xx}$, $\alpha_{yy}$ or $\alpha_{zz}$ values obtained for the set of solvent configurations from MD. However, individually derived values for the polarizabilities were not always physically meaningful. Especially in cases in which the local external electric field in a given direction is close to zero (e.g., by symmetry), small deviations from the assumed linear response of the fitted induced dipoles to the external field can lead to large uncertainties in the computed polarizability [25,26]. This can be observed even for our polarizability fitting for water, where (total) external electric fields at the polarizable oxygen center are typically high. For the 500 unique solvent configurations, we obtain a large spread in the distribution of $\alpha_{yy}$ and $\alpha_{zz}$ values (Figure 2a), with some of the values even in the nonphysical negative range.

**Figure 3:**
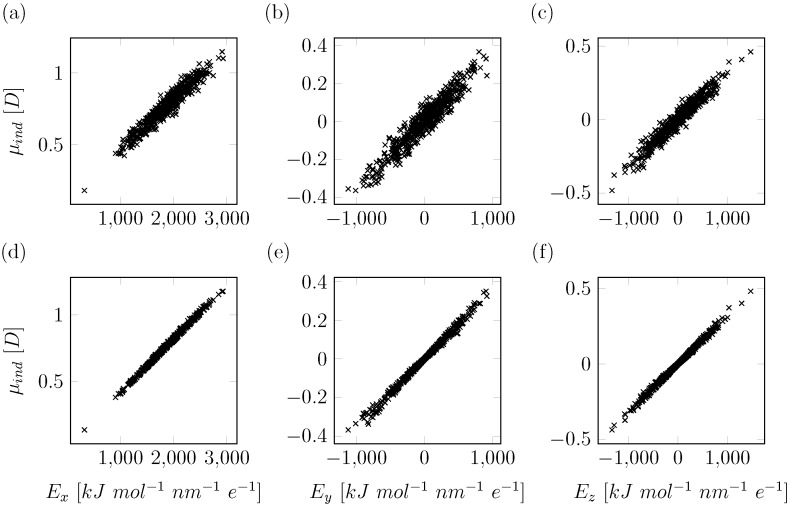
Fitted induced dipole moments ($\mu_{ind}$) in the presence of an external electric field (*E*), plotted for each separate dimensional component (*x*, *y* or *z*). In (**a–c**), the results obtained using our original method (with updated *E* calculation) are presented, where induced dipoles of the QM solute were fitted independently for each individual solvent configuration obtained from MD. The *x*, *y* and *z* components of these induced dipoles are presented in (**a**–**c**), respectively; (**d**–**f**) present the results of our consensus fitting approach, where 20 independent solvent configurations are used simultaneously in each constrained fit of the induced dipole moments. The *x*, *y* and *z* components of these induced dipoles are presented in (**d**–**f**), respectively.

In order to improve the consistency in our derived values for the polarizabilities and to narrow the distribution of values obtained for our set of solvent configurations, we introduce here two adaptations to our QM/MM approach. The first improvement is in the calculation of the effective electric fields at the polarizable center(s). Originally, a pairlist based method was used, consistent with classical molecular simulation, to compute the effective electric fields on each polarizable site. However, this can lead to inconsistencies with the list of MM charges entering the QM/MM evaluation of the MEP, as the concept of range-based cutoffs is not employed here. Hence, electric fields should be calculated using the full solvent shell that is used as Bq QM input, while inconsistencies in the employed solvent shell can have a significant influence on sites with small values (i.e., lower than 500 kJ mol^−1^ nm^−1^ e ^−1^) for the local electric field in one or multiple directions. As a result, we found that, in some cases, even the sign of the fitted multipole can change. Here, we omitted the use of a cutoff in determining the electric fields at the solute atoms, in order to be consistent with the MM solvent shells entering our QM/MM calculations. These improved electric field calculations led to a decrease in the standard deviations for the calculated polarizabilities. This is demonstrated when determining the effective molecular polarizability located at the water oxygen atom, in particular in the *y*- and *z*-directions (with relatively small values for the corresponding values of the local electric field), with standard deviations decreasing from 17.8 $\times 10^{-3}$ nm^3^ and 38.1 $\times 10^{-3}$ nm^3^ (data not shown) to 8.1 $\times 10^{-3}$ nm^3^ and 10.4 $\times 10^{-3}$ nm^3^, respectively, Figure 2a. However, negative values for the polarizability were still observed (Figure 2a) and these standard deviations still imply a significant uncertainty in the calculated polarizabilities, which may well become more pronounced in cases in which net electric fields are even lower (e.g., alkanes or other hydrophobic compounds). Note that, while the original method is noisy for small electric fields, the overall fitted induced dipoles in each dimension (*x*, *y* and *z*) do show linear response to the external electric fields. The correlation coefficient ($R^{2}$) of approximately 0.9 indicates a linear response for the range of external electric field strengths in a hydrated environment (Figure 3a–c). Based on this finding, our model does not include a damping factor of the polarizability at high electric fields, as is applied, for example, in the recently published COS/D2 model [8], which is also based on charge-on-spring polarization.

To improve fitting in cases where local electric fields are weak, our second and major adaptation to the original protocol is a redesign of the manner in which the induced dipoles are fitted after the QM/MM calculations of the MEPs. We postulate that influences beyond the external electric field, e.g., changes in the intramolecular electric field due to polarization, can broaden distributions but are hard to capture in our model. Therefore, we design here a consensus-fitting approach in which the overall optimization target is switched from a single-frame error function to a multi-frame optimization one. We do this by applying electric-field dependent constraints in a pair-wise manner between MD snapshots. These constraints are applied to each dimension of the diagonal of the polarizability tensor (Equation (10)), implemented using Lagrange multipliers and where $\alpha_{k}$ denotes the polarizability in dimension *k* (*xx*, *yy* or *zz*) and the superscripts are indices for *n* different solute-solvent configurations retrieved from MD (in the current work, *n* is set to 20):

(10) $$\alpha_{k}=\frac{\mu_{k}^{1}}{E_{k}^{1}}=\frac{\mu_{k}^{2}}{E_{k}^{2}}=\ldots =\frac{\mu_{k}^{n}}{E_{k}^{n}}.$$

To still be able to obtain a statistical estimate of the uncertainty in the determined polarizability values using Equation (10), we apply this constraint per fit on (non-overlapping) subsets of 20 randomly selected MD snapshots each, to remove configurational bias. As expected, the overall fitting error increases because the number of degrees of freedom is reduced. However, despite a twenty-fold decrease in the total number of fitting parameters, the overall squared error ($\chi^{2}$) only increases by 25% on average (Figure 2c). By enforcing linear response between a group of randomly selected frames, we obtain a better defined response to external electric fields ($R^{2}>0.99$, Figure 3d–f) when compared to using our approach based on fitting per individual MD snapshot (Figure 3a–c). It should be noted that, while this behavior is enforced within the subsets of 20 snapshots, the fact that all sets of snapshots behave in a similar manner means that we are satisfactorily capturing polarizability. As a result, distributions of $\alpha_{k}$ from this updated method are narrow (expressed by a small standard deviation) and well resolved in all dimensions, independent of the strength of the average electric field, Figure 2b. As the COS model is only implemented in an isotropic manner, we determine the isotropic polarizability with the trace of the polarizability tensor (Equation (11)):

(11) $$\alpha_{iso}=\frac{\alpha_{xx}+\alpha_{yy}+\alpha_{zz}}{3}.$$

The resulting polarizability ($\alpha_{iso}$) of 1.05 $\times 10^{-3}$ nm^3^ is slightly lower than the value of 1.1 $\times 10^{-3}$ nm^3^ determined in previous work by us and still close to the value of 1.08 $\times 10^{-3}$ nm^3^ reported by Schropp and Tavan for use in combination with a point-determined electric field [24,63]. Schropp and Tavan argued that the effective electric field <E> due to the water solvent as averaged over the approximate molecular volume of a hydrated water solute (i.e., the field that causes the actual polarization in the QM/MM calculations) is significantly lower than the point-determined $E_{O}$ at the oxygen nucleus, which is commonly used when determining induced dipole moments in molecular simulations with the COS model. This argument can explain both the lower value of effective model values for the polarizability when compared to its gas-phase estimate e.g., from quantum calculations (1.44 $\times 10^{-3}$ nm^3^) [64] and the overpolarization (in terms of too high dielectric permittivities) observed for early COS water models, for which the polarizability was set to 1.225 $\times 10^{-3}$ nm^3^ or higher [13,17]. We note that, if isotropic atomic polarizabilities are fitted on three sites (hydrogens and off-atom M site), the average molecular isotropic polarizability increases to 1.21 $\times 10^{-3}$ nm^3^ (data not shown). The fact that the molecular polarizability increases further to 1.39 $\times 10^{-3}$ nm^3^ when treating the electric field in a homogeneous manner (i.e., by applying a continuous electric field to the water solute instead of a field of explicit solvent point charges in order to determine $\phi_{solv}$ in Equation (7)) also supports the argument of Schropp and Tavan. Thus, the inhomogeneous treatment of the response to the solvent electric field may well be on the basis of the observed decrease in molecular polarizability when comparing the gas-phase reference value with our QM/MM-determined (and other) condensed-phase estimates, also considering that point-charge inclusion of the external electric field in the QM Hamiltonian cannot lead to exchange repulsion with the solvent. Our QM/MM determined value for $\alpha_{iso}$ is also close to the empirically determined polarizability of 1.04 $\times 10^{-3}$ nm^3^ in the Drude-oscillator (DO) SWM-DP model [12]. In the more recent six-site and negative DO models, this polarizability was scaled down to values of 0.97825 $\times 10^{-3}$ nm^3^ and 0.88 $\times 10^{-3}$ nm^3^ [7,16], which are lower than our QM/MM determined value.

**Figure 4:**
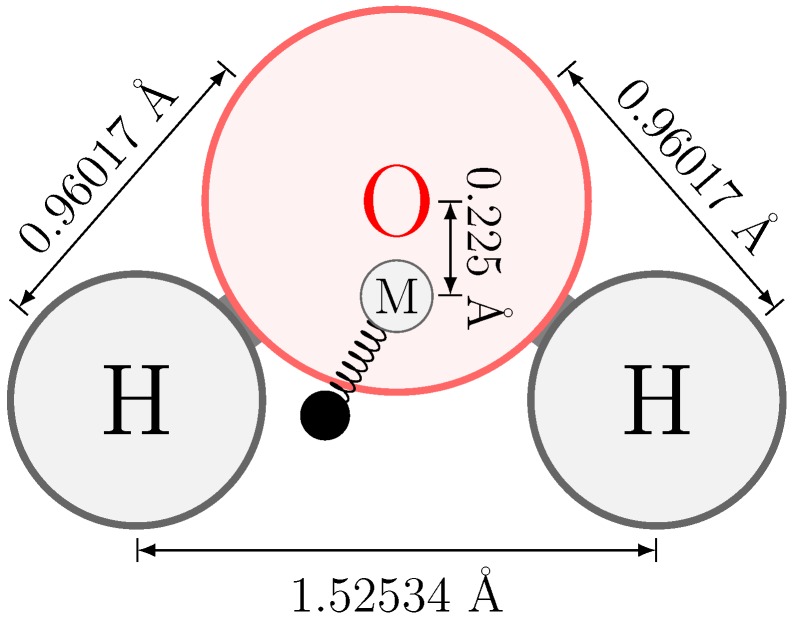
Rigid water geometry after QM optimization in the gas phase at the B3LYP/QZ4P level of theory. All bonds are constrained during simulation and the bond angle is enforced by an extra bond between the hydrogens. The location of the off-site (M) particle and its offset are included in the figure. The COS particle is attached to the M-site.

In our water model, the molecular geometry is rigid, with lengths of the (three) constrained bonds equal to those of the QM optimized gas-phase geometry, Figure 4. This rigidity allows for inclusion of an off-atom site (designated as M in Figure 4) without creating torque forces, similar to other four-site models.

**Figure 5:**
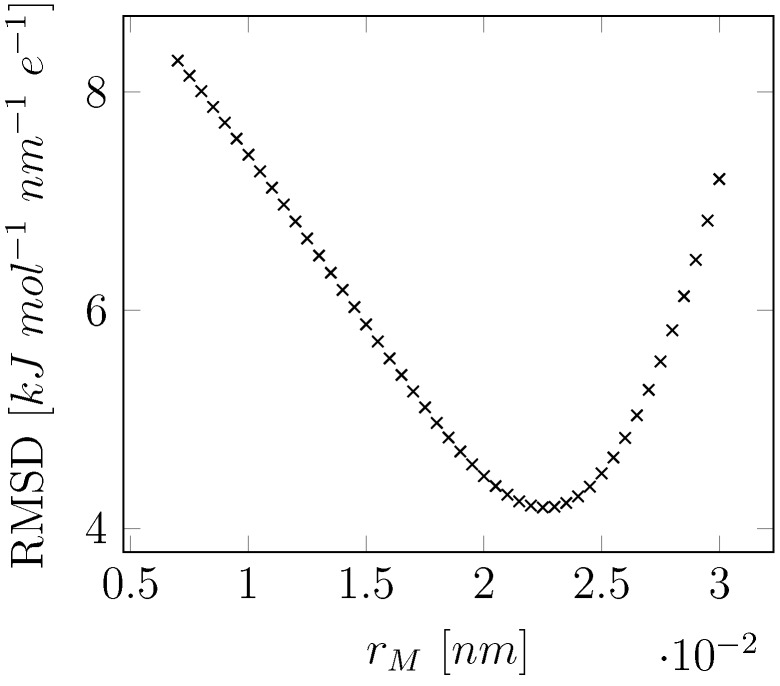
Position $r_{M}$ of the off-atom (M) site along the *x*-axis in Figure 1 versus the root-mean-square deviation (RMSD) between the partial-charge fitted and QM (B3LYP/QZ4P) estimated molecular electrostatic potential in the gas phase.

To optimize the relative position of M, a series of displacements for the off-atom site along the O-M vector was generated, ranging between 0.007 nm and 0.03 nm. For each O-M distance, a gas-phase point charge model was fitted based on the QM determined MEP in vacuum and the resulting molecular static dipole moment was evaluated. For the displacement (of 0.0225 nm) that gives a minimum in the Root Mean Square Deviation (RMSD) between the QM-determined and fitted MEP, the obtained static charge model represented the experimental static dipole well (1.856 Debye for our model versus the experimental gas-phase dipole of 1.855 Debye) [65] and was therefore chosen (Figure 5).

To describe van der Waals interactions, we use our recently proposed higher-order dispersion model that includes a $C_{8}$ term. From a QM calculation on a water solute, we directly derived atomic $C_{6}$ and $C_{8}$ coefficients as described previously by us [24] for both the oxygen and hydrogens. To preserve the simplicity of previous water models as a solvent for molecular simulation, we opted to keep a single van der Waals site. The rationale is that hydrogen-bonding is of weak covalent nature and therefore cannot be modeled solely with a simple repulsive function. Hence, the dispersion coefficients assigned to the hydrogens were re-partitioned to the oxygen site by summing the square-root of the coefficients (Equations (12) and (13)) [66]:

(12) $$C_{6,comb}^{\frac{1}{2}}=C_{6,O}^{\frac{1}{2}}+2\cdot C_{6,H}^{\frac{1}{2}}$$

(13) $$C_{8,comb}^{\frac{1}{2}}=C_{8,O}^{\frac{1}{2}}+2\cdot C_{8,H}^{\frac{1}{2}}$$

In this way, we could derive values for both $C_{6}$ and $C_{8}$ directly from quantum and XDM calculations [51], with values of 43.44 a.u. and 1201.3 a.u., respectively. These were combined with a $C_{11}$ repulsive potential as determined suitable in previous work [24]. With our QM determined static-charge model and our XDM and QM/MM values for the dispersion constants and polarizability, we could now calibrate a water model solely based on the radius of the oxygen van der Waals site. Using the definition of a van der Waals radius, $C_{11}$ repulsive constants are calculated here from the zero-point energy of the van der Waals potential energy function (Equation (1)) for which the repulsive term of the function equals the sum of the attractive terms, Equation (14). $\sigma_{ij}$ in Equation (14) denotes the sum of the van der Waals radii $r_{i}$ and $r_{j}$ for an atom *i* and *j*:

(14) $$C_{11,ij}=\left(\frac{C_{6,ij}}{\sigma_{ij}^{6}}+\frac{C_{8,ij}}{\sigma_{ij}^{8}}\right)\sigma_{ij}^{11}.$$

For the calibration of the oxygen radius, we performed a systematic single-dimension parameter scan starting from its Bondi estimate [67]. Performance of each point in the parameter scan was evaluated based on a comparison of calculated and experimental values for the pure-liquid heat of vaporization and density at 298.15 K. In this way, we found an atomic van der Waals radius for the oxygen of 0.1605 nm to be compatible with the other fitted parameters, Table 1.

With our final parameter set, both the density (ρ) and heat of vaporization ($\Delta H_{vap}$) are within 1% of the experimental value, which is similar to the performance by other COS or DO models in literature, Table 2. While the primary target of our force field optimization is a correct description of thermodynamic properties, we also evaluate model performance in terms of describing transport properties. We find that the standard diffusion constant as determined with the Einstein relation is slightly too small at 1.90 × $10^{-5}$ cm^2^ s^−1^, versus an experimental value of 2.3 × $10^{-5}$ cm^2^ s^−1^ [69]. However, Yeh and Hummer have shown that the diffusion constant depends on the simulation box size and should therefore be corrected with a term related to the shear viscosity of the liquid [61]. Correcting the diffusion coefficient based on the model’s shear viscosity (η in Table 2) results in a diffusion constant of 2.17 × $10^{-5}$ cm^2^ s^−1^, close to the experimental value.

An important validation step of any polarizable model is comparing its static dielectric permittivity with the experimental estimate. We find that our model gives satisfying results with a slightly lower value that is only 1% from experiment (Table 2 and Figure S1). The slight underestimation may be caused by treating polarizability in an isotropic manner, considering that for the (*x*) dimension with highest net electric fields, we found a slightly higher effective polarizability compared to the model’s isotropic value, cf. Figure 2b and Table 1 ($\alpha_{x}$ = 1.19 $\times 10^{-3}$ nm^3^ versus $\alpha_{iso}$ = 1.05 $\times 10^{-3}$ nm^3^). While the reference value for the pure-liquid molecular dipole moment of water has been debated for several years, there has been consensus that it is larger than the values that are typically employed in additive models [76]. Table 2 shows that our and other polarizable models also have a lower average molecular dipole moment than the value reported in Ref. [69]. Considering the treatment of electrostatics in terms of point charges and a single local inducible dipole moment in these models, their relatively low molecular dipole moment may be in line with the argument of Schropp and Tavan discussed above for the effective decrease in polarizability when going from the gas-phase reference to its QM/MM estimated condensed-phase value [63].

**Figure 6:**
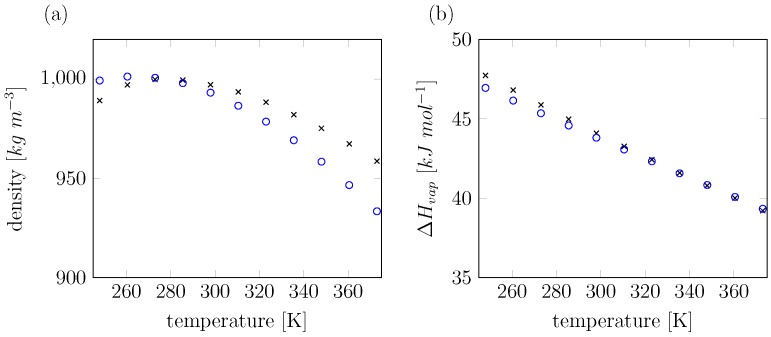
(**a**) Density and (**b**) heat of vaporization ($\Delta H_{vap}$) over a range of temperatures (248 K–373 K) for water: comparison between experimental values (black crosses) and values calculated from simulation using the water model presented in this work (blue circles). Calculated values of $\Delta H_{vap}$ are corrected for changes in vibrational modes upon evaporization, which contribute significantly as documented by Horn et al. [14].

Our model shows no further significant increase in density for temperatures below 273 K, leveling off at 1001 kg m^−3^, Figure 6a. We observe a density maximum peak at 261 K, and the heat of vaporization is well within 2% of experiment over the full range of temperatures considered (Figure 6b).

## 4. Conclusions

In this work, we redesigned a QM/MM based method to determine condensed-phase atomic polarizabilities for use in molecular simulation. For water, effective polarizabilties are lower than the gas phase estimates, consistent with earlier work by others and us. By following a consensus-fitting strategy, our redesigned approach allows for determining how large the response of induced dipole moments in explicit polarizable models should be, also in cases of low electric fields for which we previously obtained large uncertainties in the calculated polarizabilities. We used this strategy to obtain a condensed-phase value for the polarizability of water which we combined with XDM-determined $C_{6}$ and $C_{8}$ dispersion constants and a QM-derived bonded and static-charge model, to define a polarizable force field for water. Our model utilizes a $C_{6}$-$C_{8}$-$C_{11}$ potential for the van der Waals interactions and was calibrated using a single parameter (i.e., the van der Waals radius of the oxygen) while keeping all other parameters at their QM and QM/MM determined values. The final water model is to our knowledge one of the first to explicitly include higher-order dispersion, while maintaining an overall simple physical description of atomic interactions. Therefore, it could provide a basis for the parameterization of next-generation force fields that include higher-order dispersion effects. Such inclusion is of particular interest as Shaw and co-workers recently indicated that increased water–protein dispersion interactions may be required for a proper description of disordered protein states [21,22]. The resulting model shows good performance in pure-liquid properties as well as promising results in temperature response.

## Figures and Tables

**Table 1:** Force-field parameters for dispersion and electrostatic interactions. Values obtained in this work are listed together with values used in SPC and in other charge-on-spring (COS) and Drude-oscillator (DO) water models. As the form of the van der Waals potential energy function used here is different from the other models (by means of inclusion of a $C_{8}$ and $C_{11}$ term in this work), the functional form of the repulsive term is listed as $C_{x}$. Van der Waals parameters are listed using homo-dimer dispersion parameters and repulsion is listed in terms of a van der Waals radius $r_{o}$. Electrostatic parameters are listed for the atomic site of the oxygen ($q_{o}$), the atomic site of the hydrogen ($q_{h}$), the offsite position ($q_{m}$), oxygen lone pairs ($q_{l}$) and the charge on the COS particle ($q_{u}$). Damping power (*p*) and the damping field strength ($E_{0}$) are only used in the COS/D2 model. All water models (except SPC) include a single polarizable site; therefore, a molecular polarizability (α) is listed. Note that for charge assignments the values listed are the charges as written in the topology; GROMOS-style force fields (This work, SPC, COS) assign internally an effective charge of $q_{i}$-$q_{COS}$ for a polarizable site, to balance out the introduction of a large COS charge. Reference values for $C_{6}$ and α were taken from Refs. [64,68], respectively. Values for SWM4-NDP and SWM6 DO water models were taken from Ref. [7] and values for SPC, COS/G2 and COS/D2 were taken from Ref. [8].

		Expt.	This Work	SPC	COS/G2	COS/D2	SWM4-NDP	SWM6
$C_{6}$	a.u.	45.4	43.44	45.40	56.27	56.28	63.80	50.34
$C_{8}$	a.u.		1201.3					
$r_{o}$	nm		0.1605	0.1583	0.1598	0.1582	0.1592	0.1599
$C_{x}$			$C_{11}$	$C_{12}$	$C_{12}$	$C_{12}$	$C_{12}$	$C_{12}$
$q_{o}$	*e*			−0.82			1.71636	1.91589
$q_{h}$	*e*		0.539	0.41	0.5265	0.57	0.55733	0.53070
$q_{m}$	*e*		−1.078		−1.053	−1.14	−1.11466	−1.13340
$q_{l}$	*e*							−0.10800
$q_{u}$	*e*		−8.0		−8.0	−8.0	−1.71636	−1.62789
α	10^−3^ nm^3^	1.44	1.05		1.255	1.3	0.97825	0.88
$E_{0}$	kJ mol^−1^ nm^−1^ e^−1^					140		
*p*						8		

**Table 2:** Pure-liquid properties of water as calculated from a 10 ns NpT simulation at 298.15 K and 1 atm (This work). Reference experimental data (Expt.) are listed for the density (ρ) [14,70], heat of vaporization ($\Delta H_{vap}$) [14,71], diffusion constant (corrected for box size, *D*) [72], static dielectric permittivity ($\epsilon_{0}$) [33], the static ($\mu_{static}$) [65] and averaged molecular dipole moment (〈μ〉) [69], constant-pressure heat capacity ($C_{p}$) [73], thermal expansion coefficient ($\alpha_{p}$) [14,70], shear viscosity (η) [74], and isothermal compressibility ($\kappa_{T}$) [75]. Values for the diffusion constant under periodic boundary conditions ($D_{pbc}$) and the self-polarization energy ($U_{selfpol}$) are also listed. Values for SWM4-NDP and SWM6 water models were taken from Ref. [7] and values for SPC, COS/G2 and COS/D2 were taken from Ref. [8].

		Expt.	This Work	SPC	COS/G2	COS/D2	SWM4-NDP	SWM6
ρ	kg m^−3^	997	993	973	999	999	994	996
$\Delta H_{vap}$	kJ mol^−1^	44.01	43.81	43.9	43.7	44.08	43.7	44.0
$D_{pbc}$	$10^{-5}$ cm^2^ s^−1^		1.90	4.1	2.0	2.2	2.35	1.76
*D*	$10^{-5}$ cm^2^ s^−1^	2.3	2.17				2.85	2.14
$\epsilon_{0}$		78.4	77.6	64.7	96.6	78.9	78.0	78.1
$\mu_{static}$	D	1.855	1.856	2.27	1.85	1.855	1.85	1.85
〈μ〉	D	2.95	2.47	2.27	2.61	2.55	2.459	2.431
$U_{selfpol}$	kJ mol^−1^		12.5		15.9	14.4		
$C_{p}$	J mol^−1^ K^−1^	75.3	91.9	93.0	107.7	88.9		
$\alpha_{p}$	$10^{-4}$ K^−1^	2.57	3.86	9.0	7.0	4.9		
η	cP	0.89	0.72				0.66	0.87
$\kappa_{T}$	$10^{-6}$ atm^−1^	45.8	41.7	47.8	47.8	44.4		

## Supplementary Materials

The following is available online at http://www.mdpi.com/1420-3049/23/12/3131/s1. Figure S1: Averaged polarization response.
