# Supplementary material for: A QM/MM Derived Polarizable Water Model for Molecular Simulation
Source: Molecules. 2018 Nov 29;23(12):3131. doi: 10.3390/molecules23123131 (PMC6321318; doi:10.3390/molecules23123131)
Supplement: Supplementary file 1 [file molecules-23-03131-s001.pdf]

Supplementary Material

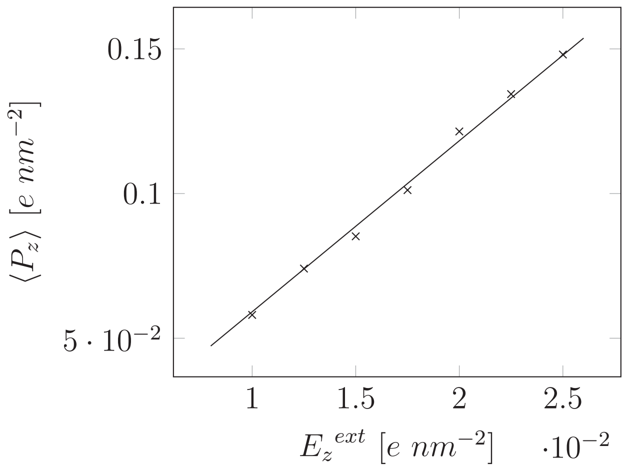

Figure S1: Averaged polarization response of our polarizable water model ($\langle P_z \rangle$) to an external electric field (${E_z}^{ext}$), used to determine the model's static dielectric permittivity by linear regression ($R^2 > 0.99$).
